# Critical misalignments in climate pledges reveal imbalanced sustainable development pathways

**DOI:** 10.1038/s41467-026-73564-5

**Published:** 2026-05-27

**Authors:** Francesca Larosa, Lamyae A. Rhomrassi, Sergio Hoyas, J. Alberto Conejero, Javier Garcia-Martinez, Fermin Mallor, Francesco Fuso Nerini, Ricardo Vinuesa

**Affiliations:** 1 School of Architecture and the Built Environment, KTH Royal Institute of Technology, Stockholm, Sweden; 2 CMCC Foundation - Euro-Mediterranean Center on Climate Change, Venice, Italy; 3 Instituto Universitario de Matemática Pura y Aplicada, Universitat Politècnica de València, Valencia, Spain; 4 Departamento de Química Inorgánica, Universidad de Alicante, Alicante, Spain; 5 FLOW, Engineering Mechanics, KTH Royal Institute of Technology, Stockholm, Sweden; 6 KTH Climate Action Center, KTH Royal Institute of Technology, Stockholm, Sweden; 7 Environmental Change Institute, University of Oxford, Oxford, United Kingdom; 8 Department of Aerospace Engineering, University of Michigan, Ann Arbor, MI 48109 USA

**Keywords:** Climate-change policy, Sustainability, Politics

## Abstract

We explore the integration of climate action and Sustainable Development Goals (SDGs) in the first two submissions of nationally determined contributions (NDCs) using an AI-based, human-validated framework. Our goal is to provide ex-ante evidence relevant to assessing policy adequacy. We find disparities in topics of interest with high-income countries emphasizing systemic challenges (health, SDG3) and low-income nations prioritizing the water-energy-food nexus (SDGs 6-7-12) and natural resource management (SDG15). We discuss what these diverging development trajectories imply for the Paris Agreement and the 2030 Agenda for sustainable development in terms of global inequality, sustainable finance flows and multilateral governance.

## Abstract (web summary)

Linking NDCs to SDGs, the authors use an AI-based, human-validated approach to reveal income-based priority gaps shaping inequality, climate finance, and global governance.

## Introduction

The 29th Conference of the Parties (COP29) in Baku closed with conflicting feelings about the future of climate action: observers agree that huge gaps remain between commitments and needs. Amid other simultaneously occurring crises, the planet is dangerously set to warm by 3.1 °C if “current policies” are implemented¹. To move from pledges to implementation, COP29 in Baku (Azerbaijan) and COP30 in Belem (Brazil) develop in continuity with its predecessor under the COP Presidencies Troika. COP28 in Dubai in December 2023 closed the first-ever Global Stocktake (GST) process: a global and comprehensive evaluation of collective progress towards meeting the 2015 Paris Agreement. The GST did not simply identify and address critical barriers and existing gaps in the climate governance landscape. It also called for a stronger alignment between climate action and sustainable development objectives^1^. Furthermore, the GST acts as a policy instrument: it informs the new rounds of NDCs (NDC3.0) providing a critical reality check and a mechanism for raising ambition^2^. While there is convergence towards the idea that the climate and sustainability concepts are inseparable^3^, stakeholders still differ in their understandings of how the 17 Sustainable Development Goals (SDGs) can be operationalized under the temperature 1.5 °C target set at COP21. The challenge ahead is complex: a mere 12 percent of SDG targets are on track to be achieved by 2030^4^, and the planet is showing concerning signs of distress as seven out of nine planetary boundaries were proven to be transgressed^5^. Progress in terms of quality and credibility in fulfilling the ten-year-old Paris target emerges from national efforts^2^. However, the speed and scale of action remain inadequate. To move along just transition pathways, fossil fuels need to be orderly phased out, while policy-makers must ensure that low-carbon technologies scale up quickly, benefiting also the most vulnerable groups^6^. Policy must foresee, assess, and avoid unintended environmental consequences of new production and consumption models^7^, ruling over the new governance of a sustainable society^8^.

Effective climate action must increasingly integrate broader sustainability objectives, minimize trade-offs and maximize synergies across dimensions to address multi-scale, concurrent challenges while sustaining momentum on climate goals (Supplementary Table 4). In line with this view, a fertile research stream has explored the interlinkages between the 17 SDGs^9^ with the explicit mandate to support the 2030 Agenda and the alignment between climate action and sustainable development^10^. Research distinguishes between synergies and trade-offs^9^ and alternatively defines them as either positive or negative influences between dimensions^11^, or as direct and indirect enablers or inhibitors in direct or indirect ways^12^. In line with this literature, we define synergy as a relation (direct or indirect) where progress on one objective or goal concretely or potentially advances progress on another objective or goal. Trade-offs occur when advancements in one goal are detrimental to another. Evidence of SDG interlinkages and their nature is gathered through automatic^13^ or manual^14^ large-scale academic literature reviews and reports^9^, which show that synergies typically outweigh trade-offs^14^. Bird’s-eye views of the SDG interaction landscape^9^, context-relevant analyses^15^, and area-specific studies^10,16^ have enriched a holistic understanding of systemic sustainability barriers. While useful, published evidence tends to focus on past or current interlinkages, falling short in supporting decision-makers with actionable knowledge. In the climate-action domain, where trade-offs have proved to be serious threats to other SDGs^10^, the analysis of win-win solutions to advance adaptation, mitigation, and sustainable development must be timely, bespoke, and accessible to policy-makers. Evidence production suffers from lack of coordinated published material: the NDCs, for example, are submitted over a long period of time. The assessment of their contribution to climate action is partial by construction and difficult to update without automatized routines capable of overcoming siloed plans for climate change adaptation and mitigation. We believe that generative artificial intelligence (genAI) tools and methods can support the systematic identification of patterns and insights to complement policy-makers’ judgement.

We provide a forward-looking outlook of the interaction between climate and other SDGs using the Nationally Determined Contributions (NDCs) as key programmatic public documents of parties’ future adaptation and mitigation plans. We contend that the analysis of the NDCs to uncover opportunities for stronger alignment between climate and sustainability agendas is an important tool to critically examine disconnected areas in light of the “common but differentiated responsibilities and respective capabilities” principles (CBDR-RC) in the UN Framework Convention on Climate Change (UNFCCC) and to inform discussions on where private capital may be more effectively delivering the Paris target under sustainable pathways. The analysis of potential misalignments between the Paris Agreement and the need to advance the 2030 Agenda for Sustainable Development is instrumental to policy coordination as advocated by the UN Expert Group on Climate and SDG Synergy^4^, the European Commission^17^, and the 6^th^ Assessment Report of the Intergovernmental Panel on Climate Change (IPCC)^18^.

The need for timely and continuous assessment of consistencies across the two intertwined agendas has been flagged by international agencies^19^, organisations^20^, and research institutions^21^. Academic studies have also tackled the type and breadth of interactions between climate and the SDGs^22^ using descriptive^10^, model-based^23^, and data-driven^24^ approaches (Supplementary Table 3). While indispensable in identifying past and present relationships across different sustainability dimensions, these works often rely on stringent assumptions and limited views regarding some SDGs (especially the social ones)^25^. We track alignment between the NDCs and SDGs contributing to the policy landscape in two ways. First, we go beyond the simple identification of climate-SDG links, and we characterize them with respect to their impact on climate adaptation and mitigation. Second, we reflect upon what current and future research can do to tackle critical misalignment to move along climate-compatible sustainable development pathways. As parties periodically review their NDCs raising their ambitions by 2035, the learning from past submissions constitutes an important building block to advance global sustainable development, negotiations over climate finance allocation, and need-based technological transfers.

### From ex-post assessment to ex-ante policy design

NDCs are programmatic documents: they uncover what countries *aim and plan to do*, rather than what countries have been doing to reach the Paris target without compromising the sustainability of their economy. Submitted to the UNFCCC, the NDCs outline national goals, priorities, and intentions to unfold climate action efforts. As such, they are political documents with clearly defined goals. While primarily focused on the climate mitigation side, Parties started including an adaptation component since the 2020-2021 submission cycle to strengthen their ambition and to widen the spectrum of potential synergies with the SDGs. The discourse analysis of the NDCs and the focus on them as narratives has been helpful to assess the credibility of pledges^26^ and the countries’ differentiated responsibilities in addressing the climate issue^27^. Since NDCs serve as a primary instrument for long-term international cooperation and negotiation, ensuring their comparability is essential. However, the diversity of these contributions presents a significant challenge for qualitative discourse analysis, complicating consistent comparisons across contexts^28^ (Supplementary Fig. 1). Most efforts look at launching tools to guide countries in building their future pledges. However, a strategic overview of the NDCs informs ex-ante policy adequacy^29^ and becomes a powerful tool to reveal if discrepancies exist and where resources shall be redirected.

**Fig. 1:**
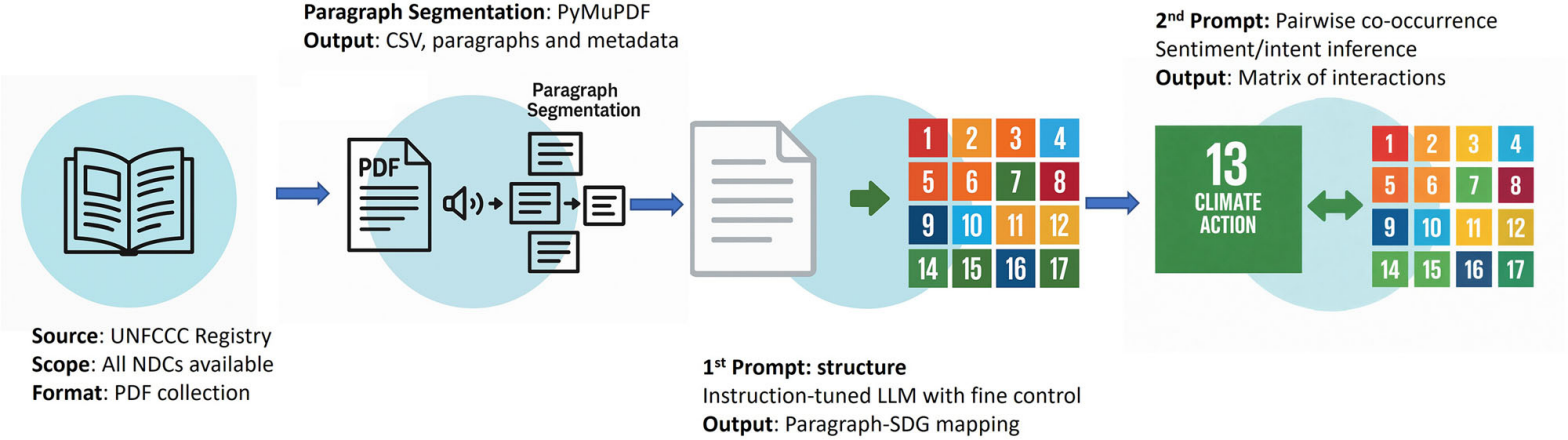
Ex-ante policy adequacy AI-assisted tool. The algorithm proceeds in four stages: (1) all NDCs are downloaded in PDF format; (2) the documents are split into paragraphs; (3) each paragraph is mapped to one or more SDGs using a general-purpose LLM; and (4) a second prompt analyzes the type of relationship between each SDG and climate action.

We implement a genAI-based procedure using the Google Research large language model (LLM) Gemini 1.0^30^ on 158 submitted NDCs (out of 195 parties by UNFCCC Registry), representing per-capita high- (50 parties), medium- (36), and low-emitting nations (72) (Supplementary Fig. 2). The embedded knowledge of both the NDCs and the SDGs in Gemini’s core training makes fine-tuning unnecessary (see Supplementary Information), reducing the environmental impact of the AI process to a minimum. We build on existing meritorious attempts in the literature^31–35^.However, our framework (Fig. 1) supports ex-ante policy adequacy design from the descriptive, but rigorous observation of different framings of risks and benefits of a climate-aligned sustainable future. Expanding on Guerrero et al. (2024)^29^, we contribute to the policy adequacy debate (i.e., the ex-ante design of appropriate measures to achieve the SDGs). We are mindful of both limits and opportunities that computational linguistic tools generate^36^ (e.g., difficult in capturing real-world implications of stated goals framed under mainstream terms), we do not infer causality, but we discuss how these findings can be integrated into better financial planning, improved governance, and capacity building.

The NDC-SDG interlinkages as a methodological approach can be transformed into a robust monitoring tool across diverse geographical and socio-economic settings and can inform comprehensive assessment while the NDC submission window is open.

### *Financial and governance implications of the Ndc-Sdg alignment*

**Fig. 2:**
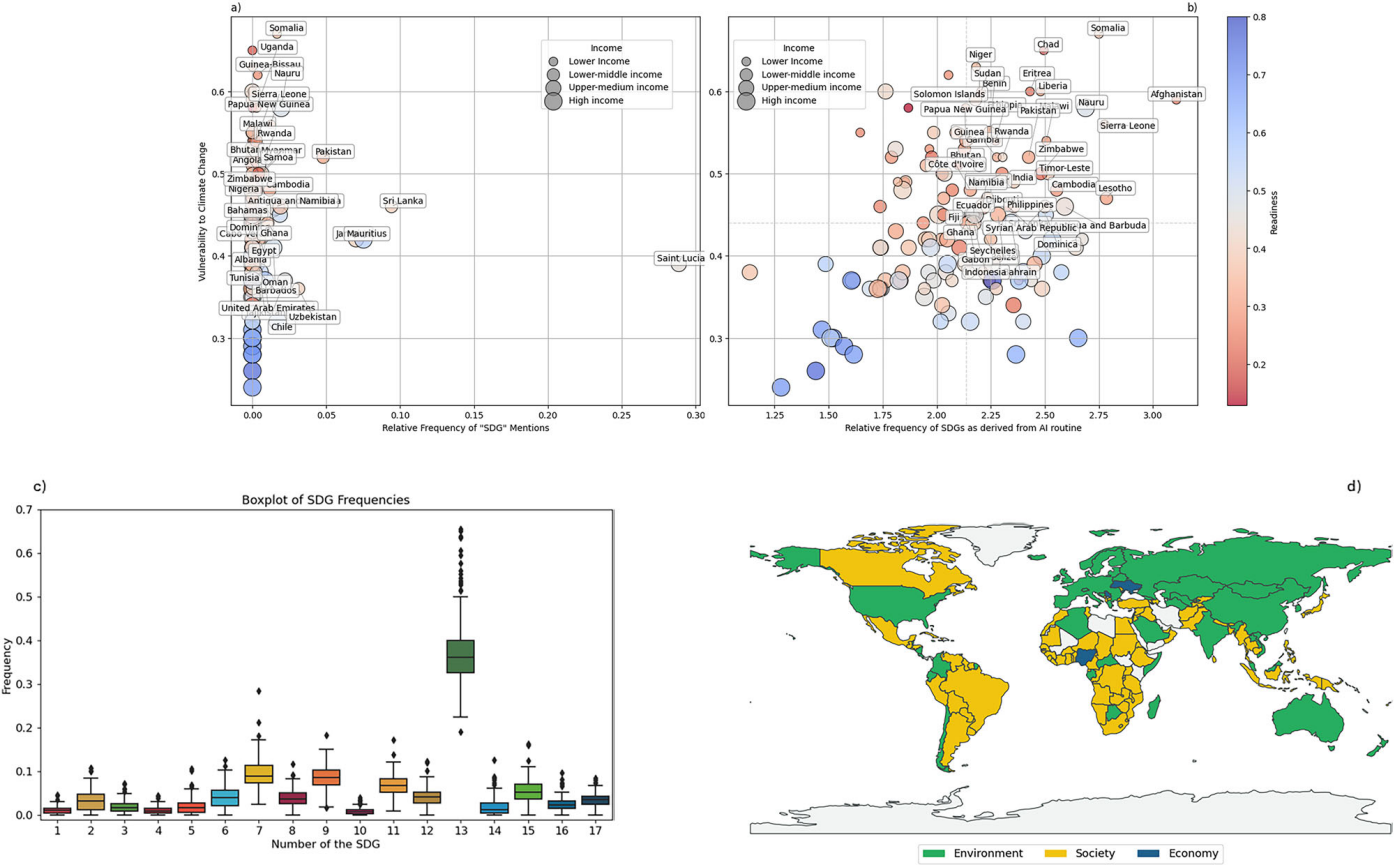
NDC-SDG classification. **a, b** The relative importance that countries assign to the SDGs in their NDCs is measured as frequency with respect to Vulnerability and Readiness (both from the Notre Dame Global Climate Adaptation Index (see Supplementary Table 2 and Supplementary Table 5 for details). **a** The search for explicitly mentioned SDGs reveals a significant alignment gap with very little text (1.01%) expanding on the two-way relationship between climate actions and sustainable development. **b** The AI-powered routine classifies the NDCs into the appropriate SDGs based on the meaning conveyed in paragraph-level texts. Over one fourth of the sample (41 countries) is in the upper right-hand quadrant. Country labels are displayed only for countries with SDG frequency >2.5. **c** The weak alignment between climate policy and the 2030 Agenda for Sustainable Development is mostly related to social aspects (education, SDG4 above all). **d** SDGs are grouped by area (environment, society, and economy) and depict a polarized world with environmental factors mostly mentioned in higher- and upper-middle-income countries.

The majority of countries considered (55.1%, 87 parties) do not explicitly mention or refer to the SDGs in their NDCs (Fig. 2a). While this misalignment is concerning for every country as the SDGs form a universal agenda, those that experience higher vulnerability and lower readiness to climate change face higher hurdles. As sustainability issues and the SDGs may be implicitly discussed or concealed in the nuances of the text, relying solely on explicit terms or keywords may not capture their full presence. Using a prompt, we classify paragraph units from the NDCs into one or more SDGs (see Supplementary Information). The ability of AI-based tools to support the identification of patterns and relationships that may not be immediately apparent to human reviewers leads to non-obvious patterns (Fig. 2b).

The positive correlation between the SDG-relevant text and vulnerability to climate change suggests that some sense of urgency is perceived especially in lower-middle (39%) or lower-income (34%) countries. Among the high-income countries making stronger reference to the SDGs in their NDCs, fossil fuel-dependent economies (e.g., Saudi Arabia, Chile) use the SDGs as a framework to plan a just transition as they need to restructure their energy systems. Once grouped as in Norström et al^37^., the SDGs depict a polarized world where SDG13 (climate action) is widely tackled by the Global North and social issues such as poverty and hunger are prioritized in the Global South. In agreement with previous literature^10^, this representation suggests that Parties submitting the NDCs tailor their policies differently and still poorly embrace a holistic view of the sustainable development agenda. This diverging position of countries concerning SDG13 also suggests that emerging and developing economies – more than high-income ones – recognize climate adaptation and mitigation as non-exclusive but integrated into a broader sustainable development domestic strategy (Fig. 2d).

#### Implication 1. Inform climate and sustainable finance architecture

**Fig. 3:**
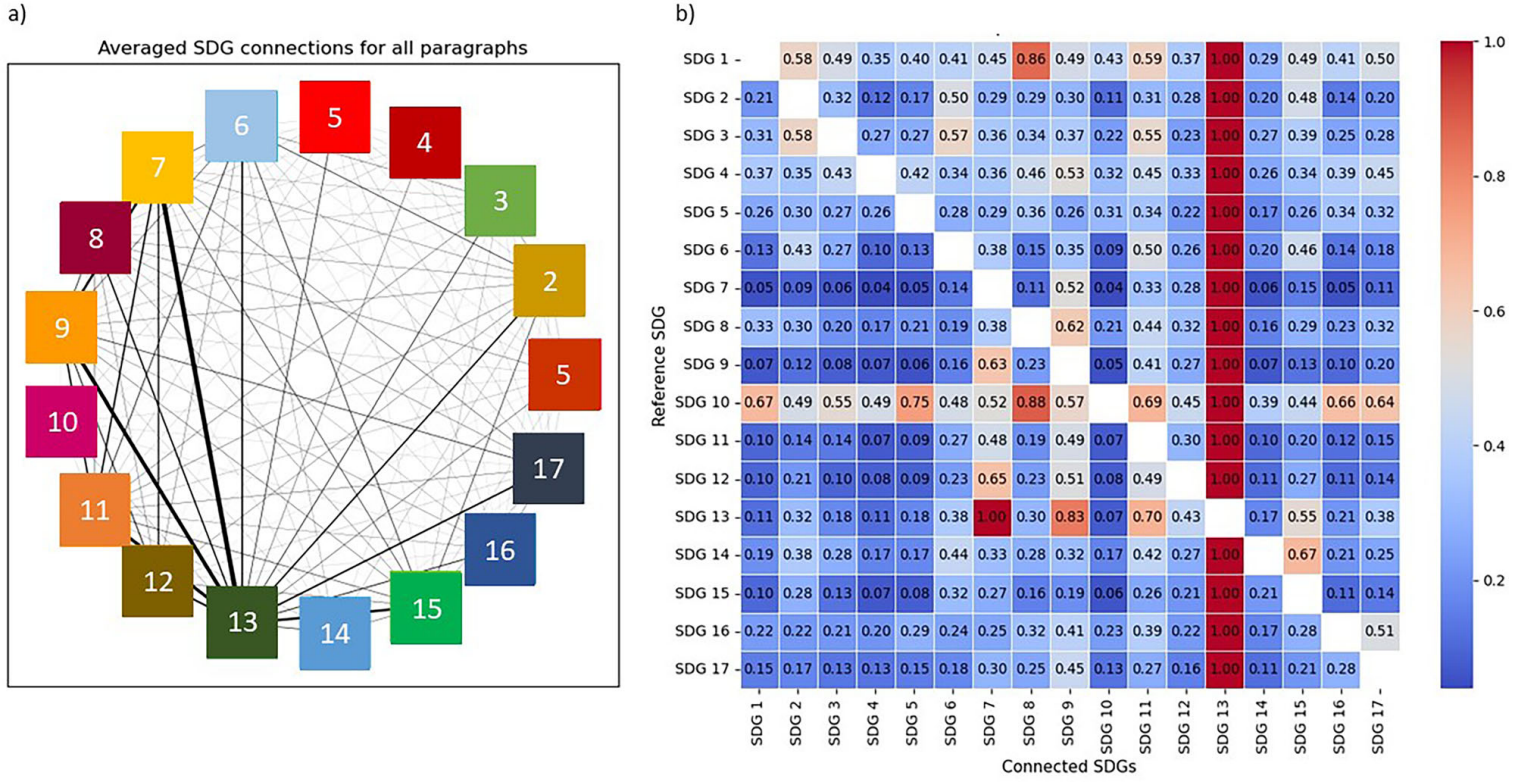
The SDG interlinkages in NDCs. **a** Connections are averaged per number of paragraphs within each NDC to ensure comparability and over the total number of countries. The *energy-infrastructure-communities* and the *infrastructures-partnerships* nexuses are the most stable, as represented by the thickness of connections. **b** The co-occurrences heat map displays the relative weight across all SDGs. Each score describes how relevant each connection is (on the X axis) with respect to each specific SDG (from the Y axis).

The identification of key SDG-NDCs links can redefine both the quality and quantity of financial flows needed to support vulnerable countries over the transition. As different intertwined challenges disproportionally affect low- and middle-income countries (LMIC), debt service often takes precedence over domestic investments to build resilience^38^. Different financial instruments (grants, concessional loans, bonds, insurance, funds, and swaps) to promote climate-compatible development agendas have generated debt distress for 79 countries, sixty of which are highly vulnerable to climate change^39^. This is partly because Debt Sustainability Analyses have consistently overlooked climate change. Even when climate change and development are integrated – for example, in the World Bank Group’s Country Climate and Development Reports – the pathways identified do not consider synergic opportunities but treat every sector as independent. However, the study of interlinkages paves the road to a more holistic outlook and can inform if and how different financial instruments can be used to mitigate or eliminate pressing challenges. Persistent co-occurrences among SDGs (Fig. 3a) are a key example of these unexplored opportunities. Forest management, afforestation, and biodiversity protection (SDG15) are priorities, especially for Sub-Saharan African Parties, but only when linked to agricultural development and land use changes. Debt-for-nature swaps may be a valid option to simultaneously address rising interest rates on debt, biodiversity loss, and climate harms. At the same time, co-occurrences reveal how widespread different issues are. For example, the NDCs acknowledge that inequality considerations exist (Fig. 3b) and it is connected to almost all SDGs. This is a pivotal issue as policies may create imbalances, especially towards the most vulnerable groups, leading to domestic conflicts and tensions (SDG16).

#### Implication 2. The NDC-SDG framework must involve relevant stakeholders

Policy measures to boost economic growth (SDG8) and sustainable urbanization (SDG11) avoid inequality consequences if a fair transition and a just distribution of resources is planned ex-ante. The just (or fair) transition, invoked by 33 Parties in their NDCs and retained also in the already submitted NDCs3.0^2^, is both an enabling condition for NDC implementation and a critical attractor of private capital. An integrated NDC-SDG framework can enhance NDC effectiveness by (i) informing the differentiated impacts of the just transition; (ii) targeting specific groups for the implementation of measures detailed in the NDCs. Operationally, the establishment of an integrated framework pursues two interrelated objectives. First, it enables policy-makers to assess whether their NDCs reflect the highest level of ambition, given national circumstances. Second, if co-developed with relevant stakeholders, it fosters durable impacts building a whole-of-society consensus. These two objectives combined simplify and harmonise the NDCs opening to the structured contribution of non-Party stakeholders (i.e., civil society organisations, financial institutions, private sector entities, local and sub-national governments) to co-develop solutions.

Our analysis reveals that there is no single codified and shared approach to include non-Party stakeholders in the NDC co-design process, but a jungle of methods (Supplementary Information). The use of an NDC-SDG framework can inform a taxonomy of actors, needs and engagement channels which work beyond national circumstances and can facilitate those multi-stakeholder monitoring and evaluation processes as already advocated by the UNFCCC and observers at COPs^40^.

**Fig. 4:**
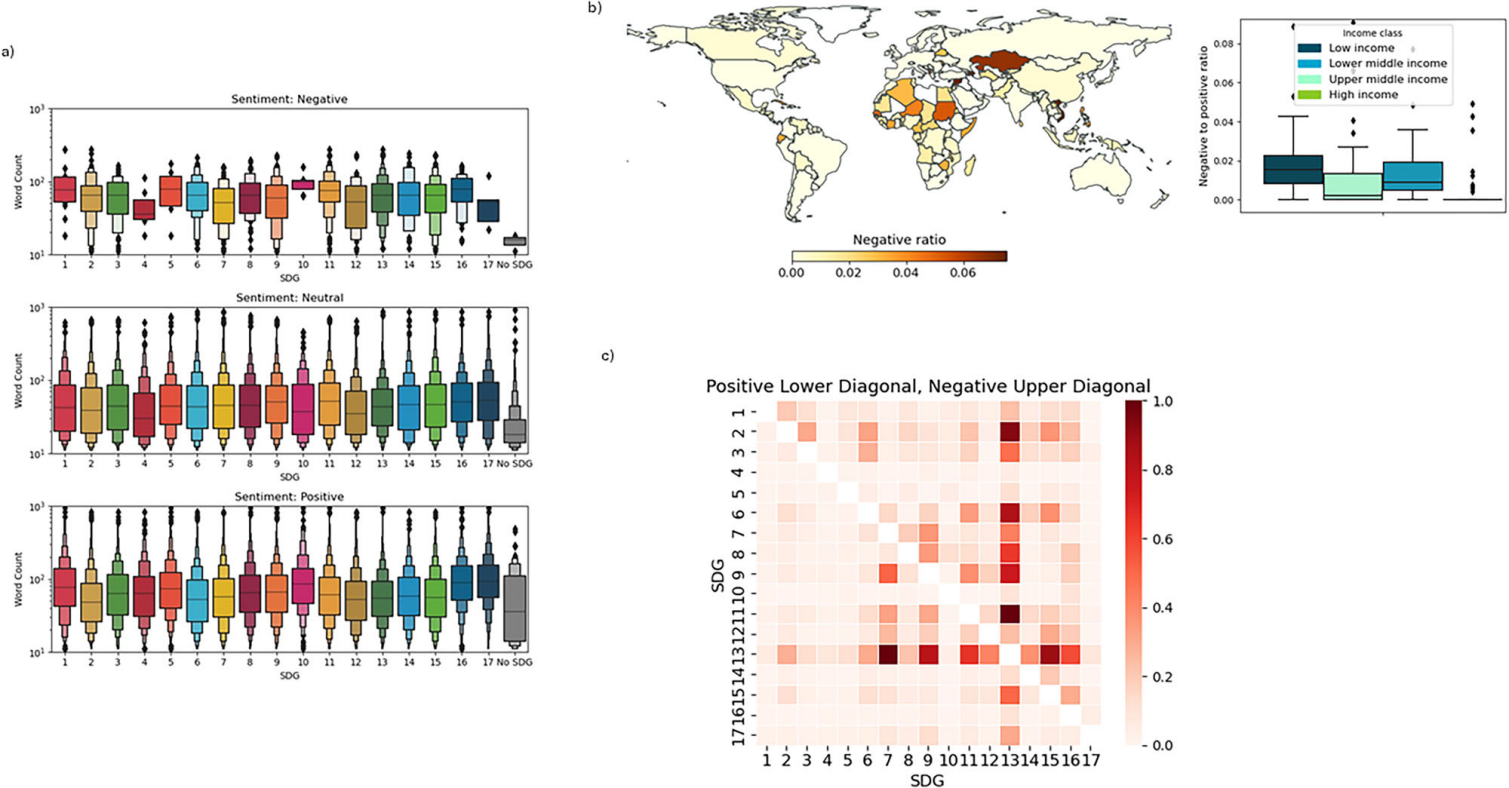
Unequal perspectives. **a** The largest variability in SDG distribution per word count and tone is visible in the negative paragraphs with socio-economic SDGs as discoverable in longer chunks. SDG4 confirms itself as poorly represented in the NDCs. **b** Parties are assigned colored per intensity of negative/positive tone ratio: the darker the color, the more negatively each party frames its NDC with respect to climate adaptation and mitigation. **c** Heatmap illustrating the normalized average frequency of SDG-to-SDG co-occurrences in positive (lower triangle) and negative (upper triangle) paragraphs. For each SDG pair, we calculate the frequency of co-occurrence per paragraph in each country, average these frequencies across all countries, and normalize the values within each sentiment class.

The alignment between NDCs and SDGs also depends on the framing, as parties unevenly present SDG interlinkages both in frequency and tone. Based on the Party’s characteristics and its related risks, links between climate policy and other sustainable development dimensions may have positive, neutral, or negative effects on domestic adaptation and mitigation strategies, hence leading to explicit synergies and trade-offs. We detect them by using a second prompt to the identified NDC-SDG connection (see Supplementary Information). The critical analysis of positive and negative spillovers describes a complex system of interactions with isolated, connected, and emerging clusters of issues and stakeholders. Only half of the text in the NDC is non-neutral (51.09%) and typically positive (synergic) outweigh negative (trade-off) links between SDG13 and other SDGs in line with existing literature^10^. As trade-offs require more elaboration (Fig. 4a), in-depth knowledge and context-relevant assessments, countries lacking adequate capacity may be disadvantaged in addressing these issues. To prevent unintended consequences triggered by negative interlinkages, support from non-governmental bodies in equipping governments with updated knowledge helps raise the ambition to assess both synergies and trade-offs. In the future, the NDCs may be used to elicit from parties which development actions contribute to or detract from climate adaptation and mitigation. This would help build in-country capacity and knowledge for the future and enhance comparability across different documents. It would also strengthen the credibility of pledges. By treating the NDC-SDG ecosystem as a complex network of interactions, several control mechanisms can be designed to evaluate needs and progress, which can differ across income groups. Table 1 provides a list of NDC-derived claims, the associated SDGs and the possible policy implications these may help forming.

**Table 1:** Examples of the link between SDG interactions and policy implementation

NDC claim	Country	Core SDG	Interaction	Policy implementation
Decrease in the share of electricity production from renewable energy due to reduced backup of balancing energy.	Moldova	SDG9	SDG7-SDG13	Supply side policy: Grid modernization and smart (flexible) grid can support intermittent energy sources. Demand side: dynamic pricing and demand response programs.
Hydroelectricity infrastructure could also be damaged during heavy rainfall events, which tend to cause unusually high amounts of sediment to flow into the rivers and dams.	Albania	SDG7	SDG9-SDG13	Maintenance of energy infrastructure in light of climate change: multi-year fund setup for adaptation.
Policy inconsistencies: Actual Electricity Act of 1956 does not allow independent power producers (IPPs) to sell to the national grid. This is a major barrier to the use of renewable sources.	Bahamas	SDG7	SDG9-SDG17	Strong energy market reform to remove or reduce barriers for incumbents in the energy market.
In line with these efforts, the Korean government will strive to improve energy efficiency, including through the distribution of energy-efficient lighting systems and appliances, and actively introduce new and renewable energy sources, including solar photovoltaic, geothermal and hydrothermal energy.	Korea	SDG7	SDG9-SDG11	To ensure a just transition, the government can implement an income-based differentiated policy structure to ensure the most vulnerable get equal access to energy efficient equipment.
When waste is not properly disposed of in landfills but in landfills or uncontrolled landfills, it becomes a problem for public health and contributes to the pollution of surface and groundwater, making it unfit for consumption.	Angola	SDG12	SDG3-SDG6	Health assessment and continuous monitoring in high-risk areas; setup of a dedicated educational program.
The main drivers of deforestation are mining, road infrastructure, urban development and agriculture.	Sudan	SDG12	SDG15	Protected areas can limit conflicts between industrial policy and biodiversity protection.

#### Implication 3. Development planning reinforces climate action

LMICs are more explicit in both synergies and trade-offs than upper-middle and high-income countries (Fig. 4b, Supplementary Fig. 3) as they reinforce the idea that planning climate actions may be constrained by financial resources availability. On the trade-offs side, LMICs highlight that within the *SDG7-SDG9-SDG11* nexus (Fig. 4c), a simultaneous expansion of infrastructure networks, energy grids and low-carbon technologies is a pre-condition for the system transition to happen. This explicit call for more holistic development planning is consistent with the idea that there is no “one-size-fits-all” solution as feasibility and effectiveness are highly heterogeneous. In fact, high-income countries tackle the positive links between SDG7, SDG9 and SDG11 more frequently than LMICs, but they anchor their narrative on benefits for climate adaptation and mitigation: they target energy efficiency in key sectors (e.g., industry, building and land transportation – Saudi Arabia) and mitigation reduction policy via electrification and fuel substitution (e.g., from oil to hydrogen in transport - Uruguay).

The framework welcomes and explores whether the identified trade-offs encompass both adaptation and mitigation. Issues related to natural resources conservation and management (SDG6, SDG15 and SDG2) and impacts on livelihoods are particularly relevant when it comes to climate adaptation in LMICs, but they constitute key assets to reduce greenhouse gas emissions (mitigation) in higher-income Parties. This new web of climate actions is shaped by a heterogeneity in skills, interests, and capabilities, and LMICs lead the way in embracing complexity across adaptation and mitigation, as well as social, environmental, and economic dimensions. Sub-Saharan African parties, for example (e.g., Sierra Leone and Togo), are among the most vocal in flagging the need to protect communities depending on freshwater bodies and fisheries from climate impacts; South Asian parties (e.g., Tajikistan) introduce the need to expand adaptation measures for rural communities through climate finance. These links are made explicit in NDCs submitted primarily after the COVID-19 pandemic, when sustainable development progress suffered from widespread halts.

### Operationalising the Ndc-Sdg alignment

**Fig. 5:**
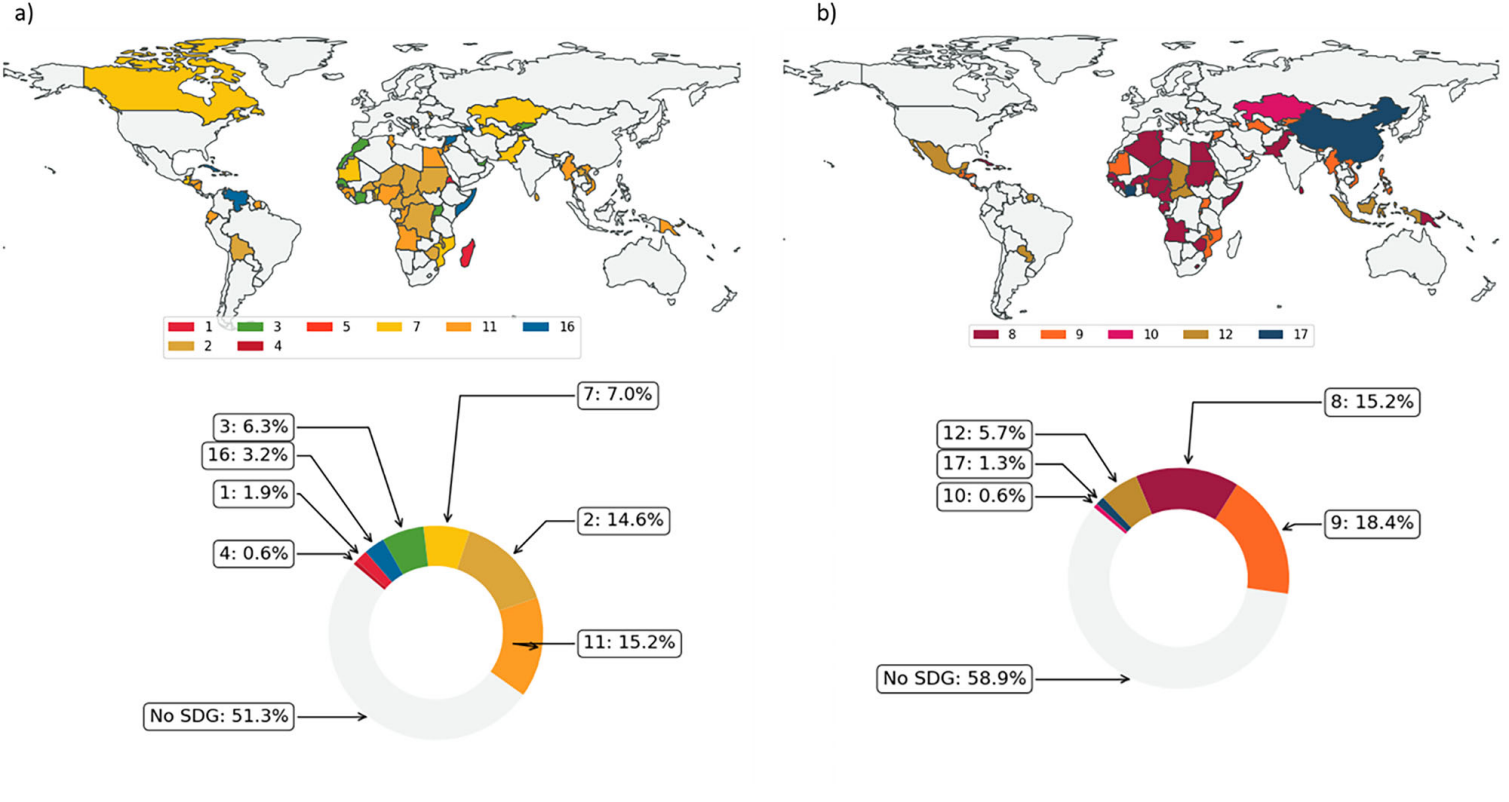
An in-depth look at trade-offs across society and the economic SDGs. **a** Societal SDGs are widespread across different income classes, mostly due to the energy transition. As climate changes, countries flag the need to train new professionals and qualified personnel to support climate adaptation and mitigation strategies (e.g., Burundi). **b** Economic SDGs reveal that macroeconomic issues are at the heart of climate action. China has called out international cooperation, highlighting the need to counteract polarization and create a decentralized governance structure. For both figures, percentages indicate the frequency of occurrence per SDG.

The existence of trade-offs between different agendas offsets synergic interdependencies between the SDGs and climate adaptation and mitigation. Strongly recognized and highlighted by LMICs (Fig. 5), these negative interactions are mitigated when cooperation, collaboration, and trusted multilateral institutions are in place. Multilateralism is at the heart of the United Nations system: adopted in 1992, the UNFCCC convenes COPs to negotiate and operationalize actions to address climate change. Multilateral institutions are typically chosen not just to host, but also to manage complex issues, as the UNFCCC process remains “the only place we have to address the rampant climate crisis”^41^. At the same time, the failures of multilateralism in delivering the promise of a prosperous and sustainable future cannot be left unaddressed. The NDCs reveal multilateralism’s weaknesses when reporting the current and future trade-offs between the Paris Agreement and the 2030 Agenda for Sustainable Development. Multilateral institutions can facilitate and propose scalable solutions through flagship and multi-year programs, and the NDCs provide evidence of the political priorities they should pursue for the benefit of all.

#### Implication 4. Multilateralism removes roadblocks to climate-aligned futures

First, a peaceful geopolitical landscape favors cooperation and collaboration to reduce and remove roadblocks to a climate-smart, sustainable development. Conflict-affected areas (e.g., Syria and Sudan) are also translating their challenges into the NDCs, revealing how relevant peaceful institutions (SDG16) are to foster climate action. Sabotages and attacks, for example, on critical infrastructure (including dams, irrigation networks, and oil and gas fields) prevent the full alignment of domestic priorities and climate change adaptation and mitigation objectives (e.g., Syria’s NDC explicitly mentions how “random extraction of crude oil […] caused a significant environmental contamination”). The current multilateral governance rooted in the UN General Assembly with a five permanent Security Council mobilized to direct and regulate over global events is showing its pitfalls. The urgency to adapt the model designed after the Second World War to the need for a hyperconnected and challenged world has never been stronger. Climate action can act as a guiding framework for such a reform: as countries share similar needs and experience comparable trade-offs, new alliances can be formed.

Second, countries report in their NDCs that tensions and trade-offs exist also within their borders between different agroecological areas: native forest coverage, for example, affects land use changes and discourages agricultural practices (i.e., the Democratic Republic of the Congo), which may also alter water cycles. Whenever coastal areas exist, climate change on agricultural land has led to overfishing, which has consequences for food security. Challenges in supporting sustainable urban communities (SDG11), climate-smart agriculture (SDG2) for all, and the adoption of low-carbon technologies (SDG7) are of primary concern, especially in low infrastructure countries’ NDCs. These unstable equilibria can be corrected and supported by strong multilateral institutions that value certain areas as universally valuable, calling the entire international community to preserve them. The Cali Fund launched during the last biodiversity summit, COP16, exemplifies this proposal: The economic resources collected from the digital use of organisms’ genetic codes will be allocated to Indigenous Peoples, either directly or through governments, in recognition of their role in safeguarding the planet’s biodiversity^42^.

Third, the NDCs further reveal other trade-off mitigation channels that multilateral institutions shall explore to promote alignment with other SDGs. Parties detail climate risks affecting households, including sea level rise, floods, and droughts. Extreme events increase the vulnerability of already vulnerable groups (e.g., Cameroon), especially in those countries where rapid urbanization processes have created chaotic developments and inadequate infrastructures (e.g., Sierra Leone). Agriculture-dependent countries, mostly in Sub-Saharan Africa (Fig. 5a), witness unpredictable and quick changes that affect yield production and income generation opportunities for rural areas. Insurance schemes are envisaged as protection mechanisms for smallholder farmers (e.g., Malawi), but very few are actually in place, and their development depends on climate finance programs, which are in turn widely supported by multilateral schemes. Multilateral institutions are the best equipped to support climate knowledge-sharing tools (i.e., climate services) and to design capacity-building programmes in national meteorological services. The Global Framework for Climate Services, for example, assists decision-makers in assessing and protecting against climate-related risks and often deploys a sector-based approach that strengthens the local economies. The integration of climate services in national weather and meteorological services can be encouraged through capacity-building activities that multilateral organisations can promote. Through its climate services toolkit, the World Meteorological Organisation is pioneering the provision of high-quality and bespoke products and services, but challenges remain^43^. The analysis of trade-offs between climate and sustainable development can act as a useful socio-economic and environmental benefit assessment tool. Those dimensions with wider mismatch can be expanded, targeting efforts and investments to improve preparedness at the local level. To achieve this goal, regional cooperation is essential^43^, and multilateral institutions act as liaison nodes in complex ecosystems of interests^44^.

Finally, the NDCs bring forward climate justice by detailing macroeconomic synergies and trade-offs of selected measures. The urgent need for a rapid and scalable energy transition (SDG 7) is a shared priority for both low and high-income countries. However, challenges are different as some countries face roadblocks in financing their infrastructure due to high cost of capital (“lack of access to cheap loans for low-carbon projects” – Kazakhstan); in some cases, the transition is perceived as a gradual diversification, but with no sudden halt of exploration activities to fight against price raises (“The Ontario government is also expanding access to natural gas across the province to help keep the cost of energy low for families, businesses and farmers” – Canada). Balance in the energy mix is also an important risk factor (e.g., Moldova) as natural disasters – such as spontaneous fires – may stress power supply without solid backup options. Given that LMICs frequently frame trade-offs between SDG7 and climate adaptation and mitigation in terms of climate impacts and shortage of technical options, climate justice must consider a consistent and stable transfer mechanism from wealthy and prepared Parties to those challenged by these hurdles. The findings underscore the critical importance of modeling both physical and transition risks associated with climate change. Scenario-based transition pathways can inform about regressive effects on specific sectors within the country. These analyses can wave those concerns related to the impacts of measures such as carbon pricing (“Social impacts, such as unemployment generated by self-charging electric vehicle stations” – Bahamas; *“The introduction of carbon pricing can lead to an increase in prices for fuel and energy resources and dependent services, and to a significant increase in inflation, which in turn can significantly worsen the welfare of the population”* - Kazakhstan). These narratives are particularly strong for high per-capita emitting countries and for fossil-dependent economies.

### Towards a coherent reporting framework for the future

**Fig. 6:**
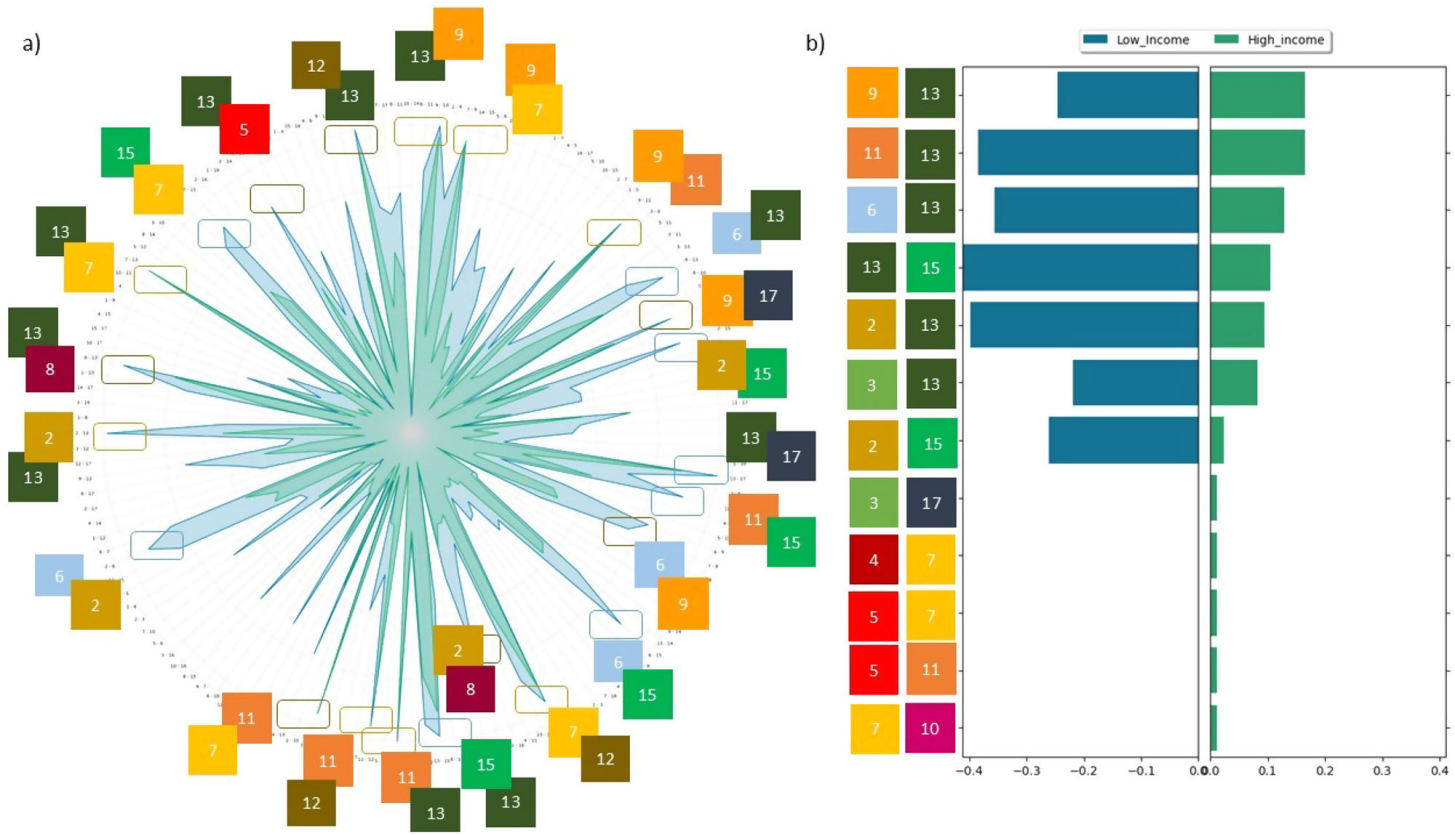
Connection stability among SDGs. Positive (**a**) and negative (**b**) stable connections (over space and time) show that high-income and low-income countries converge in tackling synergies and trade-offs, but the magnitude of their pledges differs. (**a**) Lower-income parties discuss positive links with less optimism than their high-income counterparts; rectangular shapes indicate the most mentioned interlinkages, and their color refers to environmental (blue), social (yellow), and economic (brown) dimensions. (**b**) the prevalence of SDG connections per income groups. Trade-offs present a scattered landscape with specific connections for each income group. Agriculture is the most represented sector, and water management is the top-mentioned need.

The call for multilateral institutions to promote and monitor the integration of climate and sustainable development agendas aligns with the need to identify and represent local priorities. While the responsibilities of climate change are univocally imputable to the release of greenhouse gas emissions to which many parties contributed, impacts are very heterogeneous and unevenly distributed. Therefore, countries revise their pledges according to their national priorities. Negotiations, then, make similarities, shared interests, and common goals explicit. We detect persistent synergies and trade-offs that survive across time and space, and we observe that LMICs and higher-income countries converge with important exceptions. On the synergy side (Fig. 6a), our findings align with previous literature^10^ and highlight marked opportunities between the low-carbon transition (SDG7) and climate adaptation and mitigation. If adequately planned, climate-smart infrastructure (SDG9) and novel approaches to traditional sectors (SDG12) improve alignment between the Paris Agreement and the 2030 Agenda for Sustainable Development. Adequate natural resources management is a stable synergy point in LMICs’ NDCs: being less resilient to climate impacts, the protection of natural capital fuels the local economy and the safeguard of water (SDG6), biodiversity (SDG15) directly enhances climate adaptation and mitigation.

On the trade-offs side (Fig. 6b), the NDCs devote considerable time to disaster risk reduction (SDG11) and focus their sectoral needs on transport, especially in sub-Saharan Africa. Water is an issue, especially for countries suffering prolonged droughts and poor sanitation. Water critical infrastructures (SDG6) are under threat everywhere in the world due to extreme weather events and a lack of models accounting for physical risks. Sharp differences in covering trade-offs provide an indication of diverging development trajectories in the short and medium run. The energy-infrastructure-community nexus is centered on deploying low-carbon technologies to first serve the urban population’s needs. Investments will be concentrated on realizing the transition towards clean energy systems with clear consequences on infrastructures and economic growth.

#### Implication 5. Propose public spending programmes based on synergies

The NDCs detail how national and centrally controlled budget can be mobilized to accomplish the Paris agreement without compromising sustainable development pathways. The stocktake of synergic opportunities and the analysis of existing trade-offs can unlock scalable solutions to redirect governmental expenditure. This supports (i) the reduction of the existing SDG financing gap, estimated in the order of USD 4 trillion^45^; (ii) the improvements in efficiency of public efforts; and iii) the creation of a virtuous mechanism to establish successful public-private partnerships.

As six years are left to achieve the 2030 Agenda and the “safe operating space for humanity”^46^ is rapidly closing due to anthropogenic factors, systemic approaches, and ambitious pledges are urgently needed. Instead of measuring the credibility and ambition degrees of the NDCs as in previous works^47^, examining their content sheds light on underlying political choices and leads to significant consequences. Countries differ in their core narratives and center on diverse nexuses. Hence, their development trajectories may diverge in the medium run, widening economic inequality and boosting polarization. The NDCs provide a holistic overview of climate actions and pledges, and the analysis of interlinkages with other SDGs makes critical contrasts explicit and leads to concrete policy actions (Table 2).

**Table 2:** From challenges to concrete policy implications

Finding	Observed in	Policy implication	Relevant actors
Low-income countries focus on ‘water-energy-food nexus’ (SDGs 2, 6, 7)	Low- and lower-middle-income NDCs	Climate finance should prioritize sector-integrated planning and nature-based solutions	Multilateral donors; Development banks
High-income countries emphasize ‘social SDGs’ (SDGs 3, 4, 5, 10) poorly addressing structural trade-offs	High-income NDCs	Climate policy should incorporate systemic justice metrics to address ‘just transition'	National governments, green funds
SDG4 (education) and SDG5 (gender) are consistently underrepresented across all income groups	Across all income groups	Technology transfer and skill development must be mainstreamed into gender-responsive practices	National education ministries, GCF/UNESCO
Stronger co-occurrences between SDG 7–9–11 indicate energy-infrastructure-community transition potential	All income groups	Investments should target resilient infrastructure as a systemic enabler	Climate finance institutions, City alliances, industrial players and national governments
Debt-service burden limits implementation of ambitious SDG-linked climate policies	60+ climate-vulnerable nations	Link debt relief to sustainability indicators (e.g., via debt-for-nature or debt-for-climate swaps)	IMF, World Bank; Bilateral donors
Private-sector role rarely discussed explicitly	All income groups	Introduce SDG-aligned risk reporting frameworks in future NDC cycles	Development finance institutions, ESG regulators

First, the identification of interlinkages between climate change and other SDGs can improve how international climate and sustainability funds are allocated. In agreement with the GST technical dialogues synthesis report^48^, we have found that geographic and sectoral needs are marked but no single recipe exists. An in-depth analysis of the most critical trade-offs can help redesign public economics to better address the actual needs of each country, while also enhancing monitoring efforts. As countries made explicit in their NDCs, insufficient R&D resources in lower-income economies affect infrastructures, especially after natural disasters. Meaningful implementation measures include the involvement of key actors^44^ which may connect critically underserved development and geographical areas. This evidence is more urgent than ever as the United Nations has concluded that the world is on track to warm roughly 3.1C^49^. Population dynamics in emerging economies^50^ and poorly planned development projects may lead to increased greenhouse emissions, emptying promises and efforts to raise ambitions.

Second, different narratives reveal plausible development trajectories and risks that must be detected timely. As high-income and lower-income countries differ in their core framings of climate action – per quantity, tone, and persistence – their priorities affect planning and management activities. To avoid lock-in effects, where countries become trapped in climate-vulnerable sectors and burdened by rising interest rates, a focus on core synergies and trade-offs can foster a systemic development agenda compatible with climate goals.

Policy changes over time, raising questions about revisions and monitoring plans. AI methods such as our proposed approach allow policy-makers to revisit their strategies whenever needs change. Tools such as LLMs and text-based analyses are also pivotal to collecting meaningful insights when large-scale consultations on these topics happen. They can also provide new nuances to different SDG indicators and complement large-scale data collection efforts^51^. The first 2-year-long GST process covered 252 h of meetings and more than 170.000 pages of submitted documents. The new submission rounds may benefit from human-aware and expert-reviewed LLM-based content to reveal plausible risks. COP29 reinforced the role of digital technologies and AI in promoting systemic transformation: the COP29 Declaration on Green Digital Action affirms that data-driven novel methodologies support assessment of climate impacts and support accurate standardization. The Declaration further shares the need for policies designed to integrate the “digital and low-emission transition”, placing AI at the forefront of a new dynamic interplay between digital, energy, and climate goals under the SDGs.

Finally, we find that the NDCs lack sufficient coverage of education (SDG4) and gender (SDG5) dimensions, with some exceptions in high-income countries. These pose at-risk efforts to align the 2030 Agenda with the Paris Agreement in two ways. As lower-income countries suggest in some of their submissions, qualified personnel are critically scarce to forecast disasters and to calibrate responses, making adaptation a priority. In high-income countries, physical climate risks are not adequately incorporated into public policy design, and this impacts the planning and management of critical infrastructure and urban areas. Gender-responsive policies are also at the heart of the “just transition” debate^52^ and lead to positive rebound effects on employment and economic growth^53^. Rather than embracing a hierarchical approach towards the SDGs, prioritizing some over others, we suggest that countries use their NDCs to put forward holistic measures that build on synergies to minimize trade-offs.

The Paris Agreement invites countries to unveil their “long-term strategies,” which help countries tackle their near-term plans in the next generation NDCs. The new wave of pledges will cover until 2035. Therefore, a careful and thoughtful assessment of implications for national development policy is crucial to avoid medium-term lock-in effects. With the international community calling for faster and ambitious NDCs implementation, countries will have to promote sector-relevant transformative policies while catalyzing investments from public and private actors. As we presented in this Perspective, an AI-powered, policy-relevant, and human-aware analysis can provide the timely insights needed to advance these goals. Moreover, it can highlight if inequalities across income groups and sustainability objectives exist, trying to balance efforts and available resources, as well as informing the sub-national and local policies. The current decade is the most critical to meet “the urgency of the moment”^4^: a coherent reporting framework will unlock synergies between simultaneous goals and ensure a “just, ordered and responsible”^54^ future for all.

## Supplementary information

Supplementary Information

## Data Availability

The code to reproduce this analysis can be accessed at 10.5281/zenodo.18683948 (embargoed until 18^th^ March 2026).
