# Supplementary material for: Critical misalignments in climate pledges reveal imbalanced sustainable development pathways
Source: Nat Commun. 2026 May 27;17:4719. doi: 10.1038/s41467-026-73564-5 (PMC13212587; doi:10.1038/s41467-026-73564-5)
Supplement: Supplementary file 1 — Supplementary Information [file 41467_2026_73564_MOESM1_ESM.pdf]

Data

The Perspective derives results from an artificial intelligence routine which scans climate political documents (the Nationally Determined Contributions, NDCs) through the Google Research large language model (LLM) Gemini 1.0[1].

The NDCs were downloaded from the UNFCCC NDC Registry (https://unfccc.int/NDCREG) which maintains records of active Parties' submissions in accordance with Article 4 of the Paris Agreement. Only active NDCs were downloaded from the portal excluding superseded documents from the analysis (Appendix A, Supplementary Table 5) Submissions' metadata were also included (language, status and submission date). 158 countries entered the analysis covering more than 90 percent of the global GHG emissions in per-capita terms (Supplementary Figure 1a) and submitted between 2016 and 2023 (Supplementary Figure 1b). Documents in Arab language and in non-text format (i.e., saved as images) were not included.

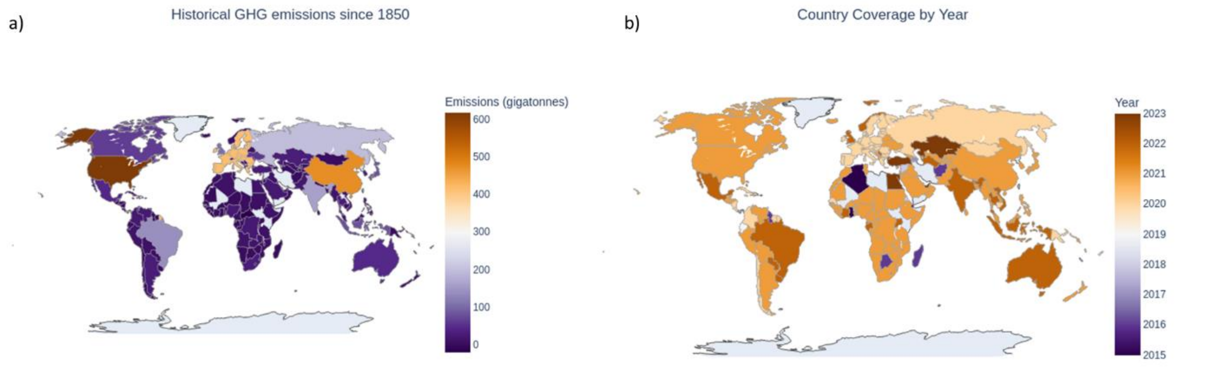

Supplementary Figure 1. Countries considered in the analysis per per-capita cumulative emissions (a) and NDC submission year (b)

The NDCs document what countries intend to do to reduce their GHG emissions within a specific timeframe and to mobilize resources to counteract on impacts[2]. Their name ("Nationally Determined Contributions") embody their goal: the NDCs "contribute" to addressing climate change according to a "nationally determined" plan given the Party's circumstances, resources and priorities (more details are included in the Glossary, Appendix A, Supplementary Table 4 This bottom-up process to advance the global effort to limit the temperature rise within 1.5°C is opposed to a top-down vision with internationally-imposed measures[3]. Countries do not follow a standard reporting template for their pledges, but they are left free to describe when they are planning to reach peaking emissions and how they intend to reduce them. This limit reduces affects comparability and transparency[4] as language and style can influence the discourses behind the NDCs. The linguistic assessment of the NDCs have been used to assess how and why countries form coalitions[5] and if ideal clusters based on similar positions shall exist[6]. The style of the NDCs also reveals different responsibilities in terms of emission reduction burden[3] and position countries differently[7] within the international arena. As emphasized in the Paris Agreement itself, "the relationship between climate change actions, responses and impacts with equitable access to sustainable development and eradication of poverty"[2] is undeniable. Therefore, the content of the NDCs reveals important constraining factors and synergic opportunities to advance domestic well-being. Research acknowledged the relationship between sustainable development and climate action in terms of policy coherence[8] and thematic alignment[9]. The SDGs are the preferred lens

through which these assessments are put forward. The variety of tools through which alignment between domestic climate plans (embodied in the NDCs) and the SDGs is assessed demonstrate the wide interest and the policy-relevance of these efforts.

To identify specific connections, the NDCs were downloaded in PDF format and split into self-contained paragraphs in a Python-based routine, Data cleaning was performed to remove *i)* figure and table titles, *ii)* short chunks (less than two words long) or with too many numerical characters (>50 percent), *iii)* sentences below 25 words repeated more than five times within the same documents. Whenever meaningful paragraphs resulted broken into separate lines, a sentence segmentation algorithm was deployed to combine different sentences using a syntactic dependency parsing technique.

We obtain a database of 31979 paragraph with metadata reported in Supplementary Table 1.

Supplementary Table 1. Database

Variable	***Type***	***Source***
Paragraph	Textual	NDCs (UNFCCC)
Number of words/paragraph	Textual	NDCs (UNFCCC)
Year of publication	Number	NDCs (UNFCCC)
Country	Textual	NDCs (UNFCCC)
Country code	Textual	The World Bank
Climate vulnerability index	Number	The Notre Dame Adaptation Index (ND-GAIN)
Climate readiness	Number	The Notre Dame Adaptation Index (ND-GAIN)
Income class	Textual (categorical)	The World Bank
Geographic area	Textual	The World bank

Methods

To check for the alignment between the NDCs and the SDGs, a two-stage prompt strategy was designed. As the SDGs are not explicitly mentioned in the NDC texts, but implicitly considered throughout the documents, the different stages respond to two sequential, but complementary goals. First, the paragraphs in the NDCs are classified to one, multiple or no SDG. Paragraphs may tackle issues simultaneously when they touch upon co-existing sustainability dimensions. Equally, paragraphs can use a sustainability-neutral language leading to no specific identified SDG. The prompt shall then be flexible enough to allow for variable SDGs per paragraph, without super imposing a pre-determined amount.

Once classified, the second prompt was designed to capture the tone of the identified paragraph with respect to climate adaptation and mitigation. The prompt was structured to assess whether a given text of the NDCs connected to one or more SDGs embodied a positive (assigned 2), neutral (assigned 1) or negative (assigned 0) meaning with respect to either one or both climate adaptation and mitigation. The purpose of this second step was to grasp what actions and recipes lead to synergies (positive) or trade-offs between the two climate and

sustainable development agendas. As the NDCs are forward-looking and programmatic documents, trade-offs were expected in fewer numbers than synergies.

The two-stage prompt was launched using a transformed-based LLM developed by Google Research, Gemini 1.0 Pro[1]. The fast-paced development of LLMs is both an opportunity and a challenge with respect to the optimal and most suitable model. At the time of the analysis, Gemini 1.0 had surpassed and advanced the status quo in large-scale language modeling according to quantitative performance assessments[1]. To further check the suitable of the model and to avoid randomness in the responses, a linguistic-grounded heuristic process assessment was designed. Gemini 1.0 was compared with GPT3.5 in a three-time classification task using three versions of the same prompt (Supplementary Table 2). Each version captured a subtle difference in meaning, pushing the model to high level sophistication. Each prompt was applied to the NDCs' paragraphs randomly ordered to check for variability in responses. *Ceteris paribus*, Gemini 1.0 proved stable within the rounds and across the different versions.

Supplementary Table 2. Alternative prompts

Version #1: quantitative assessment	"Assign the following text to the top three SDGs based on their **dominance**"
Version #2: value assessment	"Assign the following text to the top three SDGs per **relevance**"
Version #3: quali-quantity mixed	"Assign the following text to the top three SDGs based on their **prominence**"

The two prompts were designed following an iterative trial-and-error method with a climate policy expert and a programmer translating the goals to Python-based language. Seventeen versions of the two-stage prompt were tested and outputs manually screened to assess reliability. As Gemini 1.0 already has prior knowledge about the SDGs, no specific text or context around them was provided in the first stage, limiting arbitrary choices. Stage one of the strategy reads:

"Assign the following text to all relevant SDGs (strictly from the following list: 1) No poverty 2) Zero Hunger 3) Good health and well-being 4) Quality education 5) Gender equality 6) Clean Water and Sanitation 7) Affordable and clean energy 8) Decent work and economic growth 9) Industry, innovation and infrastructure 10) Reduced inequalities 11) Sustainable cities and communities 12) Responsible consumption and production 13) Climate action 14) Life below water 15) Life on land 16) Peace, justice and strong institutions 17) Partnerships for the goals). If a paragraph tackles non relevant issues with respect to any SDG, assign 0. An example of a correct output is -->
SDG (pertinent number): Name of SDG \n
Reason SDG (pertinent number): clear justification \n
SDG (pertinent number): Name of SDG \n
Reason SDG (pertinent number): clear justification ... and so on with as many pertinent SDGs."

Stage-two prompt uses the output of stage one and reads as following:

"*Use the following rules to interpret a paragraph: Consider climate adaptation as the adjustment in natural or human systems in response to actual or expected climatic stimuli or their effects, which moderates harm or exploits beneficial opportunities. Also consider, climate mitigation as an anthropogenic intervention to reduce the sources or enhance the sinks of greenhouse gas. Assign to each paragraph one and one only number between 0, 1 and 2. Assign 0 if if the paragraph explains or present an action or a set of actions which pose*

concrete risks to at least one between climate adaptation and mitigation; assign 1 if the paragraph is neutral with respect to climate adaptation and mitigation and does not express or discuss any concrete opportunity or risk for the country; assign 2 if the paragraph explains or present an action or a set of actions which pose concrete opportunities to at least one between climate adaptation and mitigation".

The output of the AI-based routine is a dense network of interactions with SDGs as nodes linked to one another in presence of at least one paragraph shared. Nodes' attributes respond to the SDG classification by Norström et al.[10] with clusters responding to economy, society and environment. Links' attributes relate to the type of node (positive, neutral or negative claim with respect to climate adaptation and mitigation). Weights are represented by the frequency of mention. Connections are represented in the form a heatmap (Figure 3c): weights are computed as bilateral connections over the raw-level maximum pairwise link. This approach overcomes the statistical bias introduced by long documents with highly mentioned connections as NDCs are all treated equally. In the global landscape, connection strengths range from a minimum of 0.04 (SDG7-SDG4) to a maximum of 1 (as for SDG13 which connects to every other SDG by construction). However, countries are not equal: they have heterogeneous socio-economic and technical characteristics which affect priorities, development challenges and ultimately pathways. Income classes derived from the World Bank Group are used to position countries into meaningful clusters (Appendix A). The classification is built on previous year's GNI per capita and allocates countries in low, lower-middle, upper-middle and high income groups. While not perfectly correlated, per-capita income classes well reflect the evolution of per-capita emissions and can then be used to describe the climate ambition need to improve country's emission profiles. Higher (high and upper-middle) income countries are more distributed across emitter classes than lower (low and lower-middle) income countries (Supplementary Figure 2a). This is also consistent with differences in SDG-SDG connection between income and emission classes. The sum of square difference between the edges of each SDG-SDG averaged connection graph, reveals that low-income connections are highly correlated with low-emitter ones and that higher income links are correlated to both middle and high emitter's (Supplementary Figure 2b).

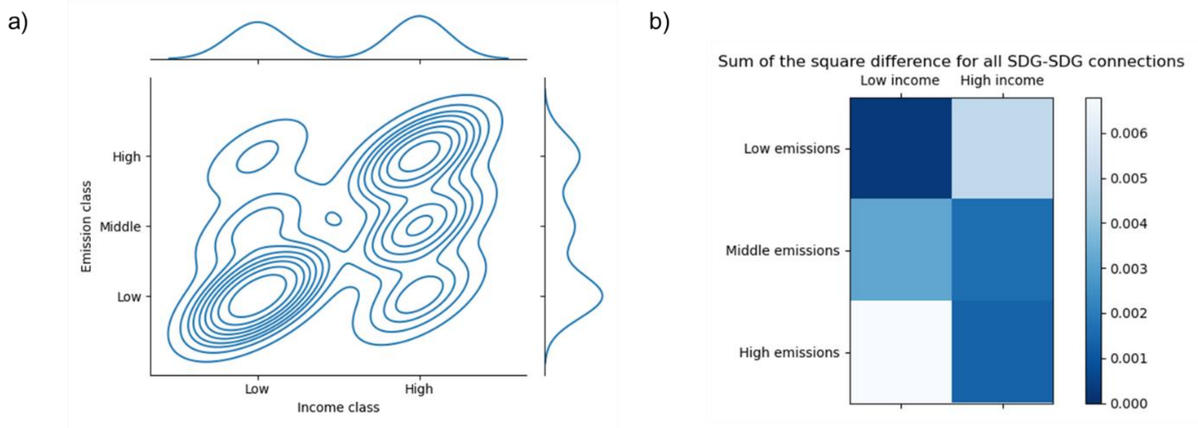

Supplementary Figure 2. Correlation between per-capita emission and per-capita income classes in absolute terms (a) and in graph-term (b)

Class-relevant interlinkages graphs are presented to compare and contrast how countries tackle the climate-sustainability link. However, some connections remain stable across the whole domain (persistent links, Figure 6) and represent the backbone of the synergy and trade-

offs opportunities. To identify these stable or persistent connections, we assign a value of 1 for each pair of SDGs in a country's NDC contribution if that connection is present, and a 0 if it is not. These values are then summed across all countries for each pair of SDGs, and the sum is divided by the total number of countries to compute the persistence metric. This method minimizes bias introduced by individual countries that may disproportionately focus on specific connections, providing a clearer picture of common interlinkages.

Evaluation of the AI routine

The AI routine produces two types of inputs: i) classification of each paragraph in one of more of the SDGs; ii) classification of each connection into a sentiment 0 (negative), 1 (neutral), 2 (positive). To perform an evaluation of accuracy of the results and to check whether assignations were correct, we followed a step-wise process.

First, we compared our results with a set of existing tools (Supplementary Table 3) which monitor the SDG-NDC alignment for diverse purposes.

Supplementary Table 3. First benchmark tools

Name	Link	Management by	Year	Description
NDC-SDG Linkages	https://www.climatewatchdata.org/ndcs-sdg	ClimateWatch	Online, updated up to May 2021	Identify potential alignment between the targets, actions, policy measures and needs in countries' Nationally Determined Contributions (NDCs) and the targets of the Sustainable Development Goals (SDGs).
NDC-SDG Connections	https://klimalog.idos-research.de/ndc-sdg/	German Institute of Development and Sustainability and Stockholm Environmental Institute	Online, updated	NDC-SDG Connections is a joint initiative of the German Institute of Development and Sustainability (IDOS) and the Stockholm Environment Institute (SEI). The research and visualisation project aims at illuminating synergies between the 2030 Agenda for Sustainable Development and the Paris Agreement, and at identifying entry points for coherent policies that promote just, sustainable and climate-smart development.

Examining the Alignment between the Intended Nationally Determined Contributions and Sustainable Development Goals	https://www.wri.org/research/examining-alignment-between-intended-nationally-determined-contributions-and-sustainable	World Resources Institute	2016	The paper explores the extent of alignment between the climate and the sustainable development agendas demonstrating that climate actions communicated in the Intended Nationally Determined Contributions under the Paris Agreement have the potential to generate mutual benefits with at least 154 of the 169 SDG targets.
Synergy Solutions for a World in Crisis: Tackling Climate and SDG Action Together	https://sdgs.un.org/synergy-solutions-world-crisis-tackling-climate-and-sdg-action-together	UNDESA	2023	A report by a group of independent experts outlining steps governments should take to maximize the impact of policies and actions by tackling the climate and sustainable development crises at the same time, creating synergies.

Second, we check whether the distribution of opportunities and risks resembles and aligns to findings from the literature. We use Fuso Nerini et al. (2019)[11] as benchmark due to the availability of a complete dataset in Supplementary materials. Third, we plot the probability distribution function (PDF) of the three sentiments per income class (Supplementary Figure 3) and we cross-checked with United Nations (2023)[12] to spot inconsistencies.

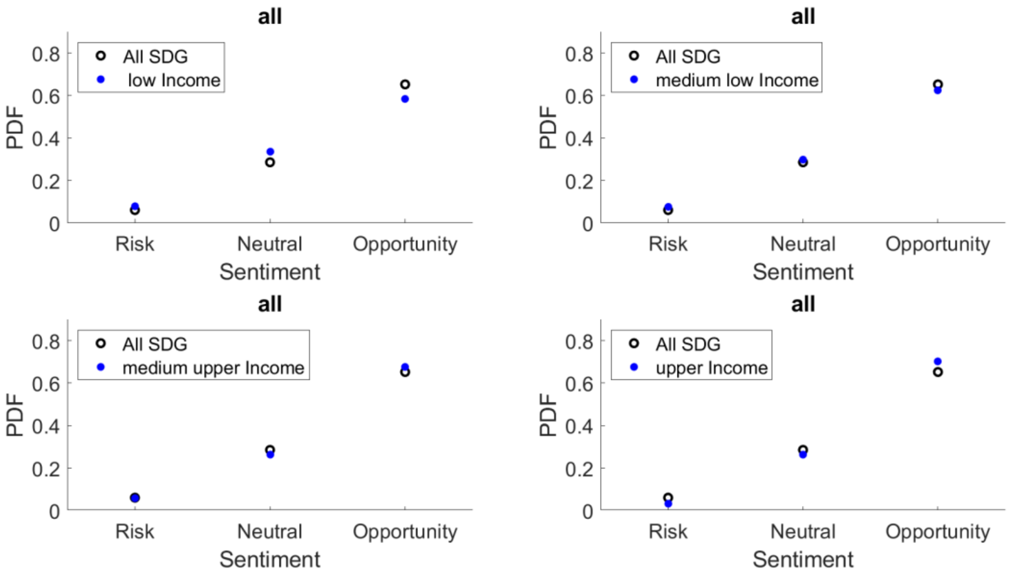

Supplementary Figure 3. PDFs of sentiment per income class

Finally, we read, screened and evaluated 5% of the total sample for both SDG assignation and sentiment. Within this sample, we find that the routine accurately predict 96% of the SDG assignation and classifies accurately the type of interlinkages in 92% of cases.

Responsible AI: ethics and limitation assessment

The use of AI tools, particularly LLMs, has inherent challenges and limitations. We describe the most relevant ones in Larosa et al. (2025)[13]. However, as the field has been evolving, we provide here further clarifications warning about potential misuses and if not carefully considered.

First, LLMs may be public (open weights) or private (closed proprietary parameters) and distributed using a free, pay-by-use or subscription-based business model. The first distinction precludes the possibility to understand processes in full and specifically how the inputs (i.e., prompts) lead to outputs (i.e., classification and sentiment). Manual checking and human expert verification protocols are essential to inform about statistical or model biases. The business model determines the willingness and opportunity to pay for the research team. As such, it may exclude vulnerable and disadvantaged backgrounds and prevent them from accessing and using private fee-based LLMs.

Supplementary References

1. Gemini Team, G. Gemini : A Family of Highly Capable Multimodal Models. *arXiv Comput. Sci.* 1–90 (2024).
2. UNFCCC. *Paris Agreement - UN Framework Convention on Climate Change* . (UNFCCC, Paris, 2016).
3. Pauw, P., Mbeva, K. & van Asselt, H. Subtle differentiation of countries' responsibilities under the Paris Agreement. *Palgrave Commun.* **5**, 86 (2019).
4. Pauw, W. P. *et al.* Beyond headline mitigation numbers: we need more transparent and comparable NDCs to achieve the Paris Agreement on climate change. *Clim. Change* **147**, 23–29 (2018).

5. Blaxekjær, L. Ø. & Nielsen, T. D. Mapping the narrative positions of new political groups under the UNFCCC. *Clim. Policy* **15**, 751–766 (2015).
6. Castro, P. National interests and coalition positions on climate change: A text-based analysis. *Int. Polit. Sci. Rev.* **42**, 95–113 (2020).
7. Mills-Novoa, M. & Liverman, D. M. Nationally Determined Contributions: Material climate commitments and discursive positioning in the NDCs. *WIREs Clim. Chang.* **10**, e589 (2019).
8. Shawoo, Z. *et al.* Increasing policy coherence between NDCs and SDGs : a national perspective. *Stock. Environ. Inst.* 1–8 (2020).
9. Janetschek, H., Brandi, C., Dzebo, A. & Hackmann, B. The 2030 Agenda and the Paris Agreement: voluntary contributions towards thematic policy coherence. *Clim. Policy* **20**, 430–442 (2020).
10. Norström, A. V *et al.* Three necessary conditions for establishing effective Sustainable Development Goals in the Anthropocene. *Ecol. Soc.* **19**,.
11. Fuso Nerini, F. *et al.* Connecting climate action with other Sustainable Development Goals. *Nat. Sustain.* **2**, 674–680 (2019).
12. United Nations. *Synergy Solutions for a World in Crisis: Tackling Climate and SDG Action Together*. *Synergy Solutions for a World in Crisis: Tackling Climate and SDG Action Together* (2023) doi:10.18356/9789213585238.
13. Larosa, F. *et al.* Large language models in climate and sustainability policy: limits and opportunities. *Environ. Res. Lett.* **20**, 74032 (2025).

Appendix A.
Glossary

Supplementary Table 4.

Term	Definition	Source
Climate action	Set of policies – at local and global level – aimed at mitigating hazardous effects of climate change. In this context, we call “climate action” the set of pledges, proposals, projects and programs which advance both climate adaptation and mitigation.	
Nationally Determined Contribution (NDCs)	“A climate action plan to cut emissions and adapt to climate impacts. Each Party to the Paris Agreement is required to establish an NDC and update it every five years”	UNFCCC NDC: https://unfccc.int/process-and-meetings/the-paris-agreement/nationally-determined-contributions-ndcs
First, updated and second submission of the NDCs	Since the launch of the Paris agreement, countries have engaged in two submission rounds. Countries interchangly distinguish between “first” and “second” or “first and “enhanced” version. In this article, we use the terminology “first” and “second” submission to distinguish between two different rounds.	UNFCCC NDC Registry: https://unfccc.int/NDCREG
Large Language Model	An AI systems which uses language as input and training material. An AI system is “a computational representation that encompasses processes, objects, ideas, people and interactions. Language models vary in language and size. AI language models are often characterised by their parameter count and layers and accuracy”	pp.22, OECD (2023)[1]
Natural language Processing (NLP)	“Computer programs and tools that automate natural language functions by analysing, producing, modifying, or responding to human texts and speech”	pp.14, OECD (2023)[1]

Supplementary Table 5. List of countries included and respective classification

Year	Country	Income group	Geography	Emission group
2016	Afghanistan	Low income	South Asia	Low emissions
2021	Albania	Upper middle income	Europe & Central Asia	Low emissions
2015	Algeria	Lower middle income	Middle East & North Africa	Middle emissions
2022	Andorra	High income	Europe & Central Asia	
2021	Angola	Lower middle income	Sub-Saharan Africa	Low emissions
2021	Antigua and Barbuda	High income	Latin America & Caribbean	
2021	Argentina	Upper middle income	Latin America & Caribbean	High emissions
2021	Armenia	Upper middle income	Europe & Central Asia	Low emissions
2022	Australia	High income	East Asia & Pacific	High emissions
2017	Azerbaijan	Upper middle income	Europe & Central Asia	Middle emissions
2022	Bahamas	High income	Latin America & Caribbean	High emissions
2021	Bahrain	High income	Middle East & North Africa	High emissions
2021	Bangladesh	Lower middle income	South Asia	Low emissions
2021	Barbados	High income	Latin America & Caribbean	Middle emissions
2021	Belarus	Upper middle income	Europe & Central Asia	Middle emissions
2021	Belize	Upper middle income	Latin America & Caribbean	High emissions
2021	Benin	Lower middle income	Sub-Saharan Africa	Low emissions
2021	Bhutan	Lower middle income	South Asia	Low emissions
2022	Bolivia	Lower middle income	Latin America & Caribbean	High emissions
2021	Bosnia and Herzegovina	Upper middle income	Europe & Central Asia	Middle emissions
2016	Botswana	Upper middle income	Sub-Saharan Africa	High emissions
2022	Brazil	Upper middle income	Latin America & Caribbean	High emissions
2020	Brunei Darussalam	High income	East Asia & Pacific	High emissions
2021	Burkina Faso	Low income	Sub-Saharan Africa	Low emissions
2020	Burundi	Low income	Sub-Saharan Africa	Low emissions
2021	Cabo Verde	Lower middle income	Sub-Saharan Africa	Low emissions
2020	Cambodia	Lower middle income	East Asia & Pacific	Low emissions
2021	Cameroon	Lower middle income	Sub-Saharan Africa	Low emissions

2021	Canada	High income	North America	High emissions
2021	Central African Republic	Low income	Sub-Saharan Africa	Low emissions
2021	Chad	Low income	Sub-Saharan Africa	Middle emissions
2020	Chile	High income	Latin America & Caribbean	Low emissions
2021	China	Upper middle income	East Asia & Pacific	High emissions
2020	Colombia	Upper middle income	Latin America & Caribbean	Middle emissions
2021	Comoros	Lower middle income	Sub-Saharan Africa	Low emissions
2021	Congo	Lower middle income	Sub-Saharan Africa	Middle emissions
2021	Congo, Dem. Rep.	Low income	Sub-Saharan Africa	High emissions
2016	Cook Island	High income	East Asia & Pacific	
2020	Costa Rica	Upper middle income	Latin America & Caribbean	Low emissions
2022	Côte d'Ivoire	Lower middle income	Sub-Saharan Africa	Low emissions
2020	Cuba	Upper middle income	Latin America & Caribbean	Low emissions
2015	Djibouti	Lower middle income	Middle East & North Africa	Low emissions
2022	Dominica	Upper middle income	Latin America & Caribbean	Low emissions
2019	Ecuador	Upper middle income	Latin America & Caribbean	Middle emissions
2023	Egypt	Lower middle income	Middle East & North Africa	Low emissions
2021	El Salvador	Lower middle income	Latin America & Caribbean	Low emissions
2022	Equatorial Guinea	Upper middle income	Sub-Saharan Africa	High emissions
2018	Eritrea	Low income	Sub-Saharan Africa	Low emissions
2021	Eswatini	Lower middle income	Sub-Saharan Africa	Low emissions
2021	Ethiopia	Low income	Sub-Saharan Africa	Low emissions
2020	European Union	High income	Europe & Central Asia	High emissions
2020	Fiji	Upper middle income	East Asia & Pacific	Low emissions
2022	Gabon	Upper middle income	Sub-Saharan Africa	High emissions
2021	Gambia	Low income	Sub-Saharan Africa	Low emissions
2021	Georgia	Upper middle income	Europe & Central Asia	Middle emissions
2015	Ghana	Lower middle income	Sub-Saharan Africa	Low emissions
2020	Grenada	Upper middle income	Latin America & Caribbean	
2021	Guatemala	Upper middle income	Latin America & Caribbean	Low emissions

2021	Guinea	Low income	Sub-Saharan Africa	Low emissions
2021	Guinea-Bissau	Low income	Sub-Saharan Africa	Middle emissions
2016	Guyana	Upper middle income	Latin America & Caribbean	High emissions
2021	Haiti	Low income	Latin America & Caribbean	Low emissions
2021	Honduras	Lower middle income	Latin America & Caribbean	Low emissions
2021	Iceland	High income	Europe & Central Asia	High emissions
2022	India	Lower middle income	South Asia	Low emissions
2022	Indonesia	Upper middle income	East Asia & Pacific	Middle emissions
2021	Israel	High income	Middle East & North Africa	Middle emissions
2020	Jamaica	Upper middle income	Latin America & Caribbean	Middle emissions
2021	Japan	High income	East Asia & Pacific	High emissions
2021	Jordan	Upper middle income	Middle East & North Africa	Low emissions
2023	Kazakhstan	Upper middle income	Europe & Central Asia	High emissions
2021	Korea Republic	High income	East Asia & Pacific	High emissions
2021	Kuwait	High income	Middle East & North Africa	High emissions
2021	Kyrgyz Republic	Lower middle income	Europe & Central Asia	Low emissions
2021	Lao PDR	Lower middle income	East Asia & Pacific	
2020	Lebanon	Upper middle income	Middle East & North Africa	Middle emissions
2017	Lesotho	Lower middle income	Sub-Saharan Africa	Low emissions
2021	Liberia	Low income	Sub-Saharan Africa	Low emissions
2017	Liechtenstein	High income	Europe & Central Asia	
2016	Madagascar	Low income	Sub-Saharan Africa	Low emissions
2021	Malawi	Low income	Sub-Saharan Africa	Low emissions
2021	Malaysia	Upper middle income	East Asia & Pacific	High emissions
2020	Maldives	Upper middle income	South Asia	Middle emissions
2021	Mauritania	Lower middle income	Sub-Saharan Africa	Middle emissions
2021	Mauritius	High income	Sub-Saharan Africa	Middle emissions
2022	Mexico	Upper middle income	Latin America & Caribbean	Middle emissions
2022	Micronesia	Lower middle income	East Asia & Pacific	
2020	Moldova	Lower middle income	Europe & Central Asia	Low emissions

2020	Monaco	High income	Europe & Central Asia	
2020	Mongolia	Lower middle income	East Asia & Pacific	High emissions
2021	Montenegro	Upper middle income	Europe & Central Asia	Middle emissions
2021	Morocco	Lower middle income	Middle East & North Africa	Low emissions
2021	Mozambique	Low income	Sub-Saharan Africa	Low emissions
2021	Myanmar	Lower middle income	East Asia & Pacific	Middle emissions
2021	Namibia	Upper middle income	Sub-Saharan Africa	Middle emissions
2021	Nauru	High income	East Asia & Pacific	
2020	Nepal	Lower middle income	South Asia	Low emissions
2021	New Zealand	High income	East Asia & Pacific	High emissions
2020	Nicaragua	Lower middle income	Latin America & Caribbean	Middle emissions
2021	Niger	Low income	Sub-Saharan Africa	Low emissions
2021	Nigeria	Lower middle income	Sub-Saharan Africa	Low emissions
2016	Niue	High income	East Asia & Pacific	
2021	North Macedonia	Upper middle income	Europe & Central Asia	High emissions
2022	Norway	High income	Europe & Central Asia	High emissions
2021	Oman	High income	Middle East & North Africa	High emissions
2021	Pakistan	Lower middle income	South Asia	Low emissions
2015	Palau	High income	East Asia & Pacific	
2020	Papua New Guinea	Lower middle income	East Asia & Pacific	High emissions
2022	Paraguay	Upper middle income	Latin America & Caribbean	High emissions
2021	Peru	Upper middle income	Latin America & Caribbean	Middle emissions
2021	Philippines	Lower middle income	East Asia & Pacific	Low emissions
2020	Russian Federation	Upper middle income	Europe & Central Asia	High emissions
2020	Rwanda	Low income	Sub-Saharan Africa	Low emissions
2021	Saint Lucia	Upper middle income	Latin America & Caribbean	Low emissions
2021	Samoa	Upper middle income	East Asia & Pacific	Low emissions
2015	San Marino	High income	Europe & Central Asia	
2021	São Tomé and Principe	Lower middle income	Sub-Saharan Africa	Low emissions
2021	Saudi Arabia	High income	Middle East & North Africa	High emissions

2020	Senegal	Lower middle income	Sub-Saharan Africa	Low emissions
2022	Serbia	Upper middle income	Europe & Central Asia	Middle emissions
2021	Seychelles	High income	Sub-Saharan Africa	
2021	Sierra Leone	Low income	Sub-Saharan Africa	Low emissions
2022	Singapore	High income	East Asia & Pacific	High emissions
2021	Solomon Islands	Lower middle income	East Asia & Pacific	High emissions
2021	Somalia	Low income	Sub-Saharan Africa	Low emissions
2021	South Africa	Upper middle income	Sub-Saharan Africa	Middle emissions
2021	Sri Lanka	Lower middle income	South Asia	Low emissions
2021	St. Kitts and Nevis	High income	Latin America & Caribbean	
2015	St. Vincent and the Grenadines	Upper middle income	Latin America & Caribbean	Low emissions
2021	State of Palestine	Lower middle income	Middle East & North Africa	
2021	Sudan	Low income	Sub-Saharan Africa	Low emissions
2020	Suriname	Upper middle income	Latin America & Caribbean	High emissions
2021	Switzerland	High income	Europe & Central Asia	Middle emissions
2018	Syrian Arab Republic	Low income	Middle East & North Africa	
2021	Tajikistan	Low income	Europe & Central Asia	Low emissions
2021	Tanzania	Lower middle income	Sub-Saharan Africa	Low emissions
2022	Thailand	Upper middle income	East Asia & Pacific	Middle emissions
2022	Timor-Leste	Lower middle income	East Asia & Pacific	High emissions
2021	Togo	Low income	Sub-Saharan Africa	Low emissions
2020	Tonga	Upper middle income	East Asia & Pacific	Low emissions
2018	Trinidad and Tobago	High income	Latin America & Caribbean	High emissions
2021	Tunisia	Lower middle income	Middle East & North Africa	Low emissions
2023	Turkey	Upper middle income	Europe & Central Asia	Middle emissions
2022	Turkmenistan	Upper middle income	Europe & Central Asia	High emissions
2022	Tuvalu	Upper middle income	East Asia & Pacific	
2022	Uganda	Low income	Sub-Saharan Africa	Low emissions
2021	Ukraine	Lower middle income	Europe & Central Asia	Low emissions
2023	United Arab Emirates	High income	Middle East & North Africa	High emissions

2022	United Kingdom	High income	Europe & Central Asia	Middle emissions
2021	United States	High income	North America	High emissions
2022	Uruguay	High income	Latin America & Caribbean	High emissions
2021	Uzbekistan	Lower middle income	Europe & Central Asia	Middle emissions
2021	Vanuatu	Lower middle income	East Asia & Pacific	Low emissions
2023	Vatican City State	High income	Europé & Central Asia	
2021	Venezuela	Upper middle income	Latin America & Caribbean	Middle emissions
2022	Vietnam	Lower middle income	East Asia & Pacific	Middle emissions
2021	Zambia	Lower middle income	Sub-Saharan Africa	Low emissions
2021	Zimbabwe	Lower middle income	Sub-Saharan Africa	Middle emissions
